# Nanotechnology Promoting the Development of Products from the Biodiversity of the Asteraceae Family

**DOI:** 10.3390/nu15071610

**Published:** 2023-03-26

**Authors:** Raíssa Mara Kao Yien, Ana Paula dos Santos Matos, Anne Caroline Candido Gomes, Denise de Abreu Garófalo, Ralph Santos-Oliveira, Naomi Kato Simas, Eduardo Ricci-Júnior

**Affiliations:** 1 Laboratory of Natural Products and Biological Assays, Department of Natural and Food Products, Faculdade de Farmácia, Universidade Federal do Rio de Janeiro (UFRJ), Rio de Janeiro 21941-902, Brazil; 2 Laboratory of Pharmaceutical Nanotechnology, Faculdade de Farmácia, Universidade Federal do Rio de Janeiro (UFRJ), Rio de Janeiro 21941-901, Brazil; 3 Laboratory of Nanoradiopharmacy and Synthesis of Novel Radiopharmaceuticals, Nuclear Engineering Institute Brazilian Nuclear Energy Commission, Rio de Janeiro 21941-906, Brazil

**Keywords:** nanoformulation, nanoemulsion, nanocapsules, herbal medicine, nanomedice, natural products

## Abstract

Biodiversity is a hallmark of the Asteraceae family. Several species are known for their pharmacological potential. The search for new substances has permeated the chemistry of natural products for years. However, the development of a final product is still a challenge. Plant extracts have physicochemical characteristics that sometimes hinder administration, requiring a formulation. In this context, nanotechnology emerges as a tool to improve the pharmacokinetic parameters of several pharmacologically active substances. Nanoemulsions, liposomes, and nanoparticles are used to carry the active ingredients and thus improve therapeutic action, especially for substances with solubility and absorption problems. This paper aimed at compiling all the studies that used nanotechnology to develop formulations from species of the Asteraceae family from 2010 to 2021 in a literature review. The search showed that nanoemulsions are the most developed formulation associated with essential oils. The use of nanotechnology promoted an improvement in the pharmacokinetic parameters of active substances.

## 1. Introduction

According to history, the use of medicinal plants to treat diseases has been a common practice throughout many human civilizations. People have looked for resources in nature with the intention of curing their ills [1,2]. Among all the potentially interesting sources for research, the *Plantae* kingdom has proven to be extremely promising over the years. The Asteraceae family, also known as the Compositae family, is the largest family of flowering plants, with a wide variety of species and a global distribution [3]. The family is divided into three subfamilies: Asteroideae, Cichorioideae, and Carduoideae, and includes a range of different plant types, including herbs, shrubs, and trees. Some of the most well-known and economically important members of the Asteraceae family include sunflowers, daisies, chrysanthemums, marigolds, and artichokes. The family is also known for its characteristic flower structure, which consists of many small flowers grouped together to form a single, composite flower head. Due to their diversity and global distribution, studies of the Asteraceae family are of great interest to botanists and ecologists. These studies can help us better understand plant evolution, diversity, and ecological interactions, as well as provide insight into the potential uses of different plant species for medicine, agriculture, and other applications [4].

Many plants in the Asteraceae family have economic, medicinal, and ornamental importance. For example, some of the most well-known members of this family include daisies, sunflowers, marigolds, and chrysanthemums, which are commonly grown for their beautiful flowers. Other members of the Asteraceae family are valued for their medicinal properties. For example, chamomile (*Matricaria chamomilla*) is a popular herb that is used for its anti-inflammatory and calming effects. Feverfew (*Tanacetum parthenium*) is also used for its anti-inflammatory properties, as well as its ability to prevent migraines. Many plants in this family contain volatile oils, which are responsible for their characteristic aromas. These oils are often used in perfumes, cosmetics, and other fragrances. Sesquiterpene lactones are another class of compounds that are found in many Asteraceae plants and are responsible for their pharmacological effects. These compounds have been shown to have anti-inflammatory, antimicrobial, and anticancer properties [4,5]. A recent review of species from the Asteraceae family shows the wide variety of pharmacologically active substance classes. Many therapeutic applications of species are attributed to their chemical compounds. Phenolic substances are characteristic, such as chicoric and chloregenic acid, and are responsible for important pharmacological effects such as antiviral, antioxidant, antimutagenic, anti-inflammatory, and radical scavenging activities. Lignans, saponins, steroids, and polysaccharides already have been described. Of the 1100 known acetylenes, molecules with biological activity, around 200 have been found in the Asteraceae tribes, including Astereae, Cynereae, Anthemideae, and Heliantheae. Each tribe has its own set of acetylene metabolites that can be used for chemotaxonomy. Although they share the same basic general chemical structure, the compounds are diverse based on two or more triple bonds. They include a range of aliphatic and cyclic structures containing sulfur, nitrogen, and oxygen. Acetylenes demonstrate various cytotoxic, anti-inflammatory, and antibiotic effects [4].

**Figure 1:**
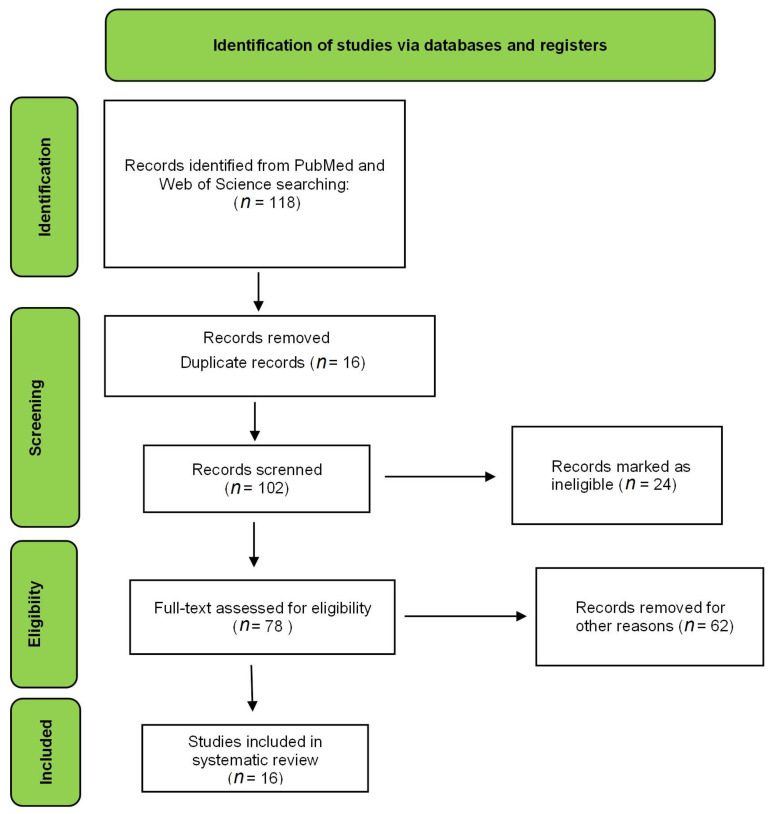
Flow diagram of the preferred reporting items for eligible articles.

Searching for new substances and therapeutic approaches always involves the technological development of a new product. Therefore, it is crucial to consider combining development technologies with basic research. In this context, nanotechnology emerges as an alternative for developing technological innovations for new pharmaceutical or cosmetic products (Figure 1). The development of nanoemulsions, liposomes, and nanoparticles has been the target of new formulations containing existing or unpublished bioactive substances. The solubility of active substances is a problem discussed by researchers and the industry [6]. Many drug-candidate substances have low water solubility and difficulty reaching relevant blood levels, being designated as classes II and IV according to the Biopharmaceutical Classification System [7]. In this context, the development of nanoformulations offers some advantages over conventional formulations, such as improved solubility, reduced toxicity, improved biological activity, and physical and chemical stability. For example, nanotechnology promotes the development of carriers used to solve the solubility problems of lipophilic actives in water. In addition, they can contribute to the delivery of the active ingredient to the target cell, enhancing the biological action [8].

Research studies on the chemistry of natural products combined with technological development are the focus of many types of research, with promising results. This review is a compilation of some of these papers that used plants from the Asteraceae family in the development of nanoformulations, going through some stages of development of a product. Additionally, we aim to understand the state of the art and the limitations of obtaining a natural nanoproduct.

## 2. Literature Search

This paper is a literature review with an electronic search in the PubMed and Web of Science databases from 2011 to 2021. The following combinations of words were used to select the studies: nanoemulsion AND Asteraceae, liposome AND Asteraceae, polymeric nanoparticle AND Asteraceae, and lipidic nanoparticle AND Asteraceae. Papers that developed a nanoformulation from extracts or natural substances isolated from species from the Asteraceae family were included. The quality of each article was evaluated according to tests and characteristics relevant to each research study, resulting in a score table. The results obtained in each database were screened and selected according to the objective of the paper. Duplicate articles, reviews, book chapters, congress abstracts, and studies with nanoformulations using particular metals, such as gold, were used as exclusion criteria.

**Figure 2:**
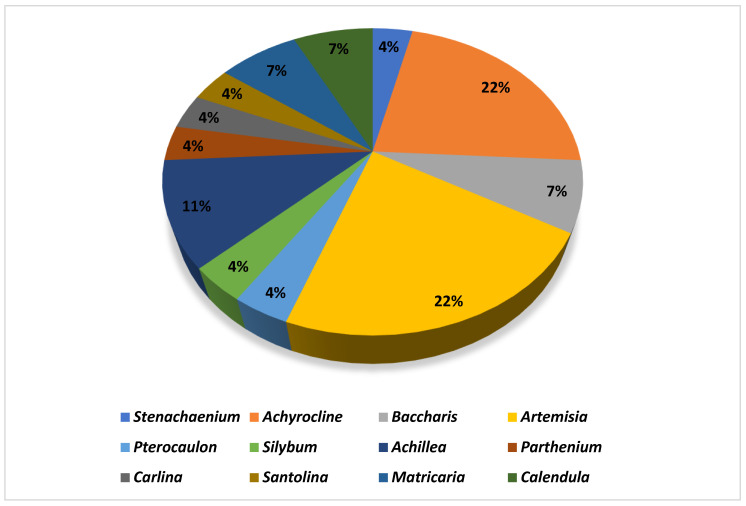
Plant genera most used in the development of nanoformulations.

The electronic search resulted in 118 articles in the PubMed database and 49 in the Web of Science database. All combinations of keywords described in the methodology were used in the search. After applying the exclusion criteria, 26 articles were used to prepare this review, as shown in Figure 2. The criteria were scored, and the total points that each article could achieve was 16 (Table 1). Evaluation of this table shows that the mean and median of the articles corresponded to 11. The article with the lowest score reached 9, and the one with the highest score obtained 14. The difference between the lowest and highest score was 5. The studies were homogeneous; consequently, the papers had similar scores. The articles with the highest score did not present cytotoxicity, stability, or in vivo studies [9,10,11]. The items that most deducted points among the papers were the cytotoxicity assay and the morphological study of the formulations.

The most developed and studied formulations are nanoemulsions [9,12,13,14,15,16,17,18,19,20,21,22,23], followed by liposomes [10,24,25,26,27], and, finally, nanoparticles [11,28,29,30,31,32,33,34]. In general, the papers have similar developments: characterization of the formulation through tests that measure particle size, PDI, and zeta potential. Electron microscopy also uses physicochemical characterization, although not in all studies [12,13,14,15,16,17,18,25,27,33]. Some papers did not develop stability studies of the formulations developed, which is an important parameter during the product development process. Only six papers conducted the stability study of the formulations tested, as shown in Table 1 [9,10,14,19,21,22,23].

**Table 1:** Score table.

References	[12]	[16]	[17]	[9]	[18]	[19]	[20]	[21]	[22]	[23]	[13]	[14]	[15]	[24]	[25]	[10]	[26]	[27]	[28]	[29]	[30]	[31]	[32]	[33]	[11]	[34]
Title	1	1	1	1	1	1	1	1	1	1	1	1	1	1	1	1	1	1	1	1	1	1	1	1	1	1
Abstract	1	1	1	1	1	1	1	1	1	1	1	1	1	1	1	1	1	1	1	1	1	1	1	1	1	1
Introduction																										
Background information	1	1	1	1	1	0	1	1	1	1	1	1	1	1	1	1	1	1	1	1	0	1	1	1	1	1
Objectives	1	1	1	1	1	1	1	1	1	1	1	1	1	1	1	1	1	1	1	1	1	1	1	1	1	1
Method																										
Phytochemistry	1	1	1	1	1	1	1	1	1	0	1	1	1	0	1	1	0	0	0	0	1	0	0	0	1	0
Voucher specimen	1	1	0	1	1	0	1	0	1	0	0	0	0	1	0	1	1	1	0	0	0	0	0	0	1	0
Formulation characterization	1	1	1	1	1	1	1	1	1	1	1	1	1	1	1	1	1	1	1	1	1	1	1	1	1	1
Stability study	0	0	0	1	0	1	0	1	1	1	0	1	0	0	0	1	0	0	0	0	0	0	0	0	0	0
Morphological study	0	0	0	1	0	1	1	1	1	1	0	0	0	1	0	1	1	0	1	1	1	1	1	1	1	1
In vitro studies	1	1	1	1	1	1	1	1	1	1	1	0	1	1	0	1	0	0	1	1	0	0	1	0	1	1
Cytotoxicity assay	0	1	0	0	0	0	0	1	0	0	0	0	1	0	0	1	0	0	1	1	0	0	1	0	1	1
In vivo studies	0	0	0	0	0	0	0	0	0	0	0	0	0	0	1	0	1	1	0	0	1	1	1	1	0	0
Therapeutic application	1	1	1	1	1	0	1	0	0	0	1	1	1	1	1	0	1	1	1	0	1	1	1	1	1	1
Statistical analysis	1	1	1	1	1	1	1	1	1	1	1	1	1	1	0	1	1	1	0	1	1	1	1	1	1	0
Results and discussion																										
Interpretation	1	1	1	1	1	0	1	1	1	1	1	1	1	1	1	1	0	1	0	1	1	1	1	1	1	1
Conclusion	0	1	1	1	1	1	1	0	0	1	1	1	0	0	0	1	0	1	1	1	0	0	0	0	1	1
Total punctuation	11	13	11	14	12	10	13	12	12	11	11	12	11	11	10	14	10	11	10	11	10	10	11	10	14	11

The criteria evaluated show studies that used commercial extracts and did not have a voucher deposited in the herbarium with botanical identification, totaling 15 articles in this situation. Some papers do not have a voucher because they use a commercial statement to justify such an absence. However, others do not describe the deposit. The phytochemical analysis is a parameter that uses analytical tools to evaluate the components in the plant extract and is essential for indicating the possible substances involved in the therapeutic effect. Most articles described the chemistry of the extracts and indicated major substances through GC-FID, GC-MS, and HPLC chromatographic analysis. Only 8 papers did not perform chromatographic analyses, indicating the main substances present [20,21,24,25,26,27,31,33].

All studies carried out in vitro or in vivo tests, but most carried out in vitro studies to demonstrate the therapeutic effect of the formulation developed, while 7 articles used in vivo tests [25,26,27,30,31,32,33].

According to Figure 2, the genera most used in the development of nanoformulations were *Achyrocline*, *Achillea*, *Artemisia*, *Calendula,* and *Bacharis. Achyrocline satureioides* was the most applied species for the development of nanoproducts. Essential oils were the most used type of extract (*n* = 13). Some studies (*n* = 5) used essential oils extracted from fresh plants, and others (*n* = 6) resorted to commercial oils to develop the nanoformulations. The most used extraction technique was maceration (*n* = 8), with ethanol as the most used extracting solvent (*n* = 5) to obtain crude extracts (Table 2). Despite being an efficient depletion technique, a large amount of solvent is spent in the process, which shows a lack of incentive for inexpensive and chemically green techniques in this context, considering that the application of green nanotechnology is a current concern.

### 2.1. Phytochemistry Information

This section is divided into the chemistry of each genre found in this research. We approach the main secondary metabolites present in the species of the genera, and Table 3 summarizes the chemical markers found in the papers included in this review.

#### 2.1.1. *Artemisia* spp.

There are more than 400 species described for the genus *Artemisia*. These species are very aromatic shrubs and can be found mainly in temperate zones of Asia, Europe, and North America. Only 9 species are cultivated in Brazil, and *Artemisia verlotiorum* L. is naturalized in the country [35]. Regarding pharmacologically active substances specific to each species herein reported, sesquiterpene lactones, monoterpenes, triterpenoids, steroids, flavonoids, and essential oils are organic classes found in *Artemisia* species. Sesquiterpene artemisinin is the major compound in *A. annua*, and artemisinic acid and alpha-thujone in *A. absinthium* [36]. In addition, *p*-allylanisole and estragole are predominant in *A. drauculus* [13,19], and β-selinene is present in *A. annua* [15]. Other active compounds can be found in this genus, such as camphor, artemisia ketone, germacrene D, 1,8-cineole, estragole, ocimene, phelladrene, and limonene [11,13,15].

#### 2.1.2. *Achyrocline* spp.

*Achyrocline* includes approximately 40 species distributed in the tropical and subtropical portions of South and Central America [37]. Macela or Marcela is the popular name of the species *Achyrocline satureioides*, a well-known plant in Brazil. The pharmaceutical industry is interested in the species, and industrial processes demand many plants. However, there is no standardized cultivation with agronomic intervention to increase the production of vegetables, which hinders industrial development. The active substances in Macela are mainly flavonoids. In addition, the main compounds are 3-O-methylquercetin, quercetin, and luteolin. Its therapeutic activities have been associated with high amounts of flavonoids, of which luteolin, quercetin, 3-*O*-methyl-quercetin, and achyrobichalcone are the most abundant. Many studies show higher activity of fractions and extracts obtained from the species than isolated substances, indicating synergism of the complex of substances [9].

#### 2.1.3. *Achillea* spp.

Genus *Achillea* comprises between 115 and 150 described species around the world. We can find naturalized species in South America, North America, Australia, New Zealand, and South Africa. Its native climate is temperate and subtropical, and its primary habitats are Iran, Turkey, Serbia, and the eastern regions of Europe [38]. *Achillea* is popularly known for its pharmacological properties and is one of the most studied and relevant botanical genera in the Asteraceae family. The characteristic substances of the species are sesquiterpenic lactones, phenolic acids (protocatechuic, vanillic, chlorogenic, ferulic and quinic acid), flavonoids (apigenin, apigeninglucoside, apigenin-rutinoside, and apigenin-neohesperioside, luteolin, vitexin, and vitexin-rhamnoside, cirsiliol, diosmetin, chrysoplenetin, chrysophanol D), lignans (sesamin), terpenic lactones (achillolid A), and alkamides (pellitorin, 8,9-Z-dehydropellitorin, anacyclin) [17,18,19,20].

#### 2.1.4. *Baccharis* spp.

*Baccharis* comprises approximately 500 American species, of which 120 are endemic to Brazil. Species of this genus are essential in folk medicine, in which several species are used in the treatment of diseases, highlighting the presence of interesting substances. More than 150 substances have been isolated, and the classes of substances already described are flavonoids, diterpenoids, triterpenoids, essential oils, phenolic substances, and coumarins [27,39]. Diterpenoids are the most prominent compounds in terms of biological activities. However, in Table 2, D-limonene (monoterpene) is the main substance present in *Baccharis reticularia*. Other minority terpenes are described: α-pinene, β-myrcene, caryophyllene, bicyclogermacrene, and spathulenol. The total of compounds identified in the essential oil is 97.2%, with 51.8% monoterpenes, 44.7% sesquiterpenes, and 0.7% diterpenes. Another species mentioned in the research is *Baccharis dracuncufolia*, with limonene, β-caryophyllene, bicyclogermacrene, and nerolidol as some of the compounds found in the essential oil.

#### 2.1.5. *Calendula* spp.

*Calendula* is represented by around 15 species (or 10–25, depending on taxonomic opinion) native to Macaronesia, North Africa, the Mediterranean region, southern and central Europe, Anatolia, Yemen, Iraq, and Iran [38]. The phytochemical profile of the *Calendula offinalis* species consists of the presence of active carotenoids, where substances such as beta-carotene, lycopene, flavoxanthin, lutein, rubixanthin, and faradiol have already been identified. We can also find flavonoids such as isorhamnetin, quercetin, narcissin, calendoflaside, rutin, neopesperidoside, and rutinoside; terpenoids such as lupeol, erythrodio, and calenduloside; coumarins (esculetin, scopoletin, umbelliferone); and essential oils (cubenol, α-cadinol, oplopanone, methyl linoleate, sabinene, limonene, α-pinene, *p*-cymene, nonanal, carvacrol, geraniol, nerolidol, t-muurolol, and palustron) [40]. In the studies carried out for this review, the chemical markers of the samples were not mentioned.

#### 2.1.6. *Matricaria* spp.

*Matricaria chamomilla* L. and *Matricaria recutita* L. are species cultivated in Brazil and are very popularly known as medicinal herbs. Recent studies show that terpenes and flavonoids are the most elementary classes, with 120 constituents described for the species. The main constituents in blue chamomile oil are chamazulene (the main substance), followed by α-bisabolol, α-bisabolol oxide A, α-bisabolol oxide B, β-bisabolene, α-farnesene, β-farnesene, cis-en-yn-dicycloether, spathulenol, and germacrene D [41]. In this review, the major substance analyzed by HPLC in the aqueous extract of chamomile flowers was the flavonoid apigenin [25].

#### 2.1.7. Other Species

Other species were used in the development of nanoformulations in this review. As they are less-used species, we grouped them all in this topic.

*Carlina acaulis* L. occurs in central and southern Europe, and the active substances are found in its roots. In the development of the *Carlina acaulis* nanoemulsion, the main compound in the root extract was carlina oxide [14]. The phytochemical composition of *C. acaulis* has been relatively poorly explored. Inulin, carlina oxide, polyacetylene, curcumene, (*E*, *Z*)-α-farnesene, β-sesqui-phellandrene, 1,8-cineole, phenolic compounds, and pentacyclic triterpenes have been reported. The main flavonoids are represented by *C*-glycosyl flavones such as orientin, homoorientin, and vitexin, which can also be found in the species. Moreover, isoschaftoside, apigenin 7-*O*-glucoside, and apigenin have been detected. The *C. acaulis* herb is also a rich source of chlorogenic acids [42].

*Parthenium hysterophorus* is indeed a noxious weed and is known to cause significant harm to human health and livestock. The plant’s allelopathic properties have been researched and found to be useful in controlling plant pests and diseases. The phytochemical profile is diverse, with various chemical compounds present, such as sesquiterpene lactones, pseudoguaianolides, flavonoids, phenolic acids, volatile oils, alkaloids, lipids, steroids, proteins, tannins, and metallic elements [23,43]. Sesquiterpene lactones and pseudoguaianolides are two major classes of compounds found in *P. hysterophorus* that are responsible for its allelopathic effects. These compounds are known to inhibit the growth of other plant species and have been used in the development of natural herbicides [23,43]. Flavonoids and phenolic acids, on the other hand, are known for their antioxidant properties, which can provide protection against free radicals. Additionally, *P. hysterophorus* is rich in several amino acids, particularly glycine, and proline, which play an essential role in protein synthesis and plant metabolism. Moderate levels of other amino acids, such as alanine and lysine, are also present, which are important for the synthesis of enzymes and other proteins [23,43]. The presence of metallic elements, such as iron and magnesium, may also contribute to the plant’s physiological processes. Overall, the diverse phytochemical profile of *P. hysterophorus*, particularly its allelopathic properties and rich amino acid content, makes it an intriguing and potentially valuable plant species for agricultural and pharmaceutical applications. However, its noxious weed status and harmful effects on human health and livestock must also be taken into consideration [23,43].

There are approximately 26 species described for the genus *Pterocaulon.* The plants are usually used as infusions and decoctions. Coumarins are abundant and widely distributed, and they are considered the main constituent of the genus. Flavonoids, terpenes, and polyacetylenes are also present. Coumarin 5-methoxy-6,7-methylenedioxycoumarin is found in the *P. balansae* species. Both extracts and compounds isolated from these species have been tested in vitro for antiviral, antiparasitic, antifungal, and insecticidal activities, among others [20,44].

*Silybum marianum* L. has great economic importance due to its main active substance. Silymarin is commercialized in capsules and extracts to treat liver problems. Other polyphenolic flavonoids are found in the species: silibinin, taxifolin, silydianin, isosilybin A, isosilybin B, silychristin, and isosilychristin [45].

*Stenachaenium megapotamicum* B. is an ornamental plant that occurs in southern Brazil, mainly in Santa Catarina and Rio Grande do Sul. It is also found in the southeastern region of São Paulo. Its phytochemical profile and therapeutic applications are still being studied, and there is limited information about the species. The essential oil was analyzed, and oxygenated monoterpenes (thymol), sesquiterpenes (β-bisabolene), and oxygenated sesquiterpenes were described. Fokienol was the main substance present in the essential oil. However, nerolidol, spathulenol, eudesmol, and cadinol were also found [12].

### 2.2. Biological Activities

Table 4 shows the diversity of the therapeutic applications of the species found in the Asteraceae family from the development of a nanoformulation. The therapeutic applications described in this review corresponded to acaricidal, antibacterial, antinociceptive, antiviral, antifungal, and larvicidal activity.

The species from the genus *Achyrocline* showed more results in studies using their extracts. In different models, several studies have reported their biological activities such as anti-inflammatory, hepatoprotective, antioxidant, immunomodulatory, antimicrobial, trypanocidal, and photoprotective. The antioxidant, larvicidal, and antiviral activities were tested from nanoemulsions with promising results. The anthelmintic, antioxidant, and cytotoxic activities were evaluated from nanocapsules. The results of the antioxidant activity of *Achyrocline satureoides* nanoemulsions promoted topical permeation of the quercetin-enriched extract [17]. The development of a hydrogel nanoemulsion containing *A. satureoides* promoted photoprotection against UV rays, and the TAR assay showed an effect 4.5 times more potent than the positive control (Trolox) [9]. Antiviral activity against the herpes virus was developed and tested from nanoemulsions. It showed a decrease in viral load in the in vitro topical assay [16]. *A. satureoides* nanocapsules also evaluated hematological parameters [30], liver cytotoxicity [32], and ROS reduction in the hearts of rats infected with *Trypanosoma evansi* [31]. All three papers show an improvement in the parameters evaluated, showing the potential of species *A. satureoides* when associated with nanotechnology formulations.

*Artemisia* was the second-most-used genus for the development of nanoformulations. Species of this genus have medicinal properties such as analgesic, antioxidant, anti-inflammatory, antimicrobial, antitumor, antipyretic, antiparasitic, and cytotoxic. Another essential therapeutic application is their action in the treatment of malaria due to the antimalarial activity of artemisinin. In this review, *Artemisia* species were tested to assess antibacterial, larvicidal, antitumor, and antinociceptive potential. Nanoemulsions were developed with *A. draucunlus* and *A. annua* essential oils to evaluate larvicidal and antimicrobial activities. The *A. draucunlus* nanoemulsion showed larvicidal activity against the larval stage of *Anopheles stephensi*, with 82% mortality [19], as well as antibacterial activity against *Escherichia coli*, *Listeria monocytogenes*, *Salmonella enteritidis*, *Shigella dysenteriae*, and *Staphylococcus aureus*; in addition, it presented antioxidant activity. The *A. annua* nanoemulsion showed antibacterial and antifungal activity against *E. coli*, *S. aureus*, *P. aeruginosa*, *S. pyogenes*, *S. pombe*, *Candida albicans*, *C. tropicalis*, *C. dubliniensi,* and *C. krusei* [15]. The MIC90 results for each assay are presented in Table 3. Another formulation developed was the polymeric liposome with *A. afra* essential oil to evaluate antibacterial activity. The nanoformulation showed activity against *E. coli* and *C. albicans* [24]. For the evaluation of the cytotoxic and antinociceptive activities, polymeric nanoparticles were developed. The *A. absinthium* extract showed cytotoxic potential in breast cancer cells, and *A. aucheri* combined with bupivacaine had a synergistic effect on reducing pain in vivo [11,33].

*Achillea* is the third most used genre in the review. It is popularly known for its pharmacological properties and is one of the most studied and relevant botanical genera in the Asteraceae family. The essential oil of the species has antibacterial, antifungal, herbicide, diuretic, anti-inflammatory, and neuroprotective activity [46]. The *A. fragrantissima* and *A. santolina* essential oils were used to prepare nanoemulsions and tested for acaricidal activity against *Tyrophagus putrescendiae*. The assay result showed LC50 values of 4.7 and 9.6 µL/L for *A. fragrantissima* and *A. santolina*, respectively. Incorporating the essential oil in a nanoformulation increased acaricidal activity and suggested an alternative to controlling this plague that harms agricultural production [22]. Another paper tested the antinociceptive effect with the combination of *A. millefolium* extract and *Origanum vulgare* in a liposome formulation. The synergistic combination of the extracts in the formulation resulted in a reduction of the pain threshold in vivo with 66% pain inhibition [26].

Other genera were less used, and we will describe their therapeutic applications below. In this review, the *Baccharis draucunculifolia* extract was incorporated into liposomes and evaluated for anti-inflammatory activity in vivo. The results showed a reduction in the production of inflammatory cytokines such as interleukins 6 and 1β, as well as tumor necrosis factor α [27]. Another research study developed a *Baccharis reticularia* nanoemulsion and evaluated the larvicidal potential of *Aedes aegypti* larvae at stage 4. The larvicidal assay showed a 34% reduction in LC50 at 24–48 h. The mortality rate agrees with the literature that used other *Baccharis* species [18]. The essential oils obtained from *Baccharis dracunculifolia* and *Baccharis uncinella* native plants are used in the perfume industry, providing an exotic aroma to various perfumes. In addition, many studies on the biological activities of these species highlight their allelopathic, antioxidant, antimicrobial, cytotoxic, and anti-inflammatory effects.

*Calendula officinalis* has been described for topical applications in cosmetology and dermatology, and its therapeutic applications involve healing processes. A number of studies have suggested its use for ecchymosis and skin eruptions, as well as for treating eczema. In Brazil, the flower tea is used as an expectorant and in anemia, in addition to its common use as an anti-inflammatory in traditional medicine. Because of the therapeutic potential, research groups have developed nanoparticles to deliver *C. officinalis* extracts with anti-inflammatory, healing, and cytotoxic properties in MCF7 adenocarcinoma cells [28,29]. Incorporation of the marigold extract into a nanoparticle increased the therapeutic effect of the plant and offered a therapeutic alternative in a promising formulation.

*Matricaria chamomilla* sin. *Matricaria recutita*, as well as marigold, is described in Brazilian herbal medicine. Chamomile is a medicinal plant used in the form of infusions. Literature reports indicate that chamomile is used for stomach pain, irritable bowel syndrome, and insomnia, as well as for bactericidal, relaxing, and acaricidal activities. In vivo studies have reported anxiolytic, cholesterol-lowering, antimutagenic, antidiabetic, and healing properties [41]. Liposomes with chamomile extract were developed to analyze the anti-inflammatory activity in a clinical trial with patients with dermatitis. The study showed that the liposomal formulation was more effective than the non-liposomal ones, thus being an alternative to corticosteroid treatment [25]. Another study developed nanocapsules and evaluated the leishmanicidal activity of chamomile. The results showed a reduction in cytotoxicity due to the encapsulation of the extract, in addition to inhibition of the promastigote and amastigote forms of *Leishmania amazonensis* [34].

*Carlina acaulis* is a plant from central Europe used in folk medicine as a diuretic, tonic, antimoral, and antimicrobial, as well as for gastrointestinal and respiratory tract problems. In this review, *C. acaulis* essential oil was incorporated into a nanoemulsion to test the larvicidal activity of the *Lobesia botrana* insect in its first larval stage. The results showed promising activity with LC50 and LC90 values of 9.04 and 17.70 µL/m, respectively [14].

*Parthenium hysterophorus* is a weed known for being extremely aggressive to agricultural production and also for being a bitter weed. Because of this natural characteristic of the species, the crude extract was incorporated into a nanoemulsion to evaluate its herbicidal activity against *Diodia ocimifolia*. The test results showed total inhibition of in vitro twinning of seeds at a concentration of 5 g/L when compared to the crude extract at a concentration of 10 gL^−1^ [23].

*Stenachaenium megapotamicum* is a species with a phytochemical profile and therapeutic application that is still poorly studied; therefore, there is little information about the species. Only floristic surveys on this genus have been published, and there are few reports about widespread use as an anti-inflammatory, antithrombotic, and anticoagulant. In this review, the essential oil presented foekinol as the predominant substance. It was used as the oil phase of the nanoemulsion developed against *Epidermophyton floccosum* and *Trichophyton rubrum*, revealing antifungal activity in skin diseases [12].

*Satolina insularis* is an endemic species in Italy. The inflorescences in this region are popularly used as vermifuges and repellents. The leaves are used as sedatives, febrifuges, and antitussives. In this review, the essential oil of the species was used in developing liposomes as nanocarriers of active substances. The nanoformulation achieved good skin permeation results in human keratinocytes [10].

*Silybum marianum* is well known for its hepatoprotective properties. However, it also has antibacterial, cardioprotective, anticancer, antidiabetic, and dermal protective properties, among others. This review incorporated the crude extract into a nanoemulsion to assess oral absorption. The in vitro results showed good permeation using cells from the PAMPA and CACO-2 cell models for oral delivery [21].

Even with similar biological activities, it is difficult to compare the results because the assays and the objectives of the studies are different. Among the antioxidant activities described, it was impossible to obtain a correlation between the results because the tests performed were not the same across the studies. However, species *A. satureoides* was present in three papers, being developed in two nanoemulsion systems [9,17] and one nanocapsule system [31], demonstrating its antioxidant potential. Among the nanosystems, the TBARS assay was performed in two studies. One paper demonstrated the antioxidant activity of the *A. satureoides* nanoemulsion in photoprotection. In contrast, the antioxidant activity of the nanocapsules was tested in infected mice with *T. evansi*, and ROS reduction was observed.

The larvicidal activity analysis was performed by three studies using nanoemulsions against *Aedes aegypti*, *Anopheles,* and *Lobesia botrana*. LC50 was performed in two studies. The best result was LC50 = 9.04 µL/mL of the *Carlina acaulis* nanoemulsion against *Lobesia botrana* [14]. The test against *Anopheles* only evaluated the mortality rate, not the LC50.

Regarding antibacterial and antifungal activity, the *A. annua* and *A. draucunlus* nanoemulsions were tested against *E. coli*, *S. aureus*, *P. aeruginosa*, *S. pyogenes*, *S. pombe*, *C. albicans*, *C. tropicalis*, *C. dubliniensis*, *C. krusei*, *Listeria monocytogenes*, *Salmonella enteritidis,* and *Shigella dysenteriae*. The best results with the lowest MIC assay were from the *A. annua* essential oil incorporated in the nanoemulsion. The *A. afra* essential oil liposome was tested against *E. coli*, *P. aeruginosa*, *S. aureus*, and *C. albicans* with poor MIC results when compared to the nanoemulsion ones. The most promising paper was with the nanoemulsion from the *A. annua* essential oil.

*Achillea millefolium* (liposome) and *Artemisia aucheri* (nanoparticle) [33] were tested for antinociceptive activity, and *A. millefolium* liposomes obtained the best result for pain inhibition (66%) in an in vivo formalin assay [26]. For the anti-inflammatory activity, the tests performed are not comparable. *Baccharis dracunculifolia* liposomes were tested in rats with a decrease in inflammatory cytokines [27], and chamomile liposomes [25] were tested topically in a clinical trial where a reduction in the inflammatory process on the skin was observed.

The anticancer activity of *Calendula officinalis* nanoparticles [28] and *Artemisia absinthium* [11] demonstrated activity against cancer cells. However, only the *A. absinthium* study described the IC50 of the cytotoxicity assay, revealing greater research robustness.

### 2.3. Nanoformulation Information

Table 5 presents the parameters evaluated for each nanoformulation developed. Nanoemulsions were the most prepared formulation type [8,9,10,11,12,13,14,15,16,17,18,19,20], followed by nanoparticles [26,27,28,29,30,31,32,33] and liposomes [21,22,23,24,25]. A nanoemulsion is a nanometric dispersion of water in oil stabilized by a surfactant. The particle sizes are smaller than 1000 nm. However, some authors cite droplet sizes of 1–100 nm. In this review, the particle sizes ranged from 11.2 to 295.6 nm, within the values discussed in the literature. As observed in the papers, this size results in a pharmacological improvement effect. Using essential oils is a good tool for this type of development, as oil works as the oily phase of the system. This advantage justifies why essential oils were more often used as extracts in this review.

The most used method to develop the nanoemulsions was the low-energy technique. A total of 8 papers [8,9,13,14,15,16,17,18] employed low energy to obtain the nanoemulsions, and the other 5 articles [10,11,12,19,20] resorted to high energy. A nanoformulation can be obtained with high or low energy. Spontaneous emulsification is known to be faster and less expensive. In contrast, high-energy techniques can be acquired from high-pressure homogenizers or ultrasound generators. This energy supplied to the system increases the deforming forces, making it possible to break the droplets into smaller sizes. Thus, more energy and/or surfactant are required to obtain smaller droplets, making this method industrially unfavorable. It is necessary to use a method that uses lower amounts of energy and low-energy emulsification methods [47].

A total of eight articles developed nanoparticles, one of them incorporating the nanocapsules in a gel [33]. The methods for obtaining nanoparticles involved warm microemulsion technique, deposition of preformed polymer, free radical emulsion polymerization, free radical mechanism, and sonication. The particle size ranged from 80 to 801 nm. Nanoparticles can be divided into nanocapsules and nanospheres. Nanocapsules are nanostructures composed of an oily “core” surrounded by a polymeric monolayer and are very useful in encapsulating hydrophobic drugs or active ingredients such as sunscreens [48]. Nanocapsules and vesicular nanosystems both involve confining a drug within a cavity or core, and both can have a polymeric membrane surrounding the drug. Nanocapsules are a type of core–shell system, which means they have a core that contains the drug and is surrounded by a shell or membrane [48]. The shell is typically made of a polymer, which can protect the drug from degradation and control its release profile. One difference between nanocapsules and vesicular nanosystems is that vesicular nanosystems typically have a lipid membrane, while nanocapsules have a polymeric membrane. Additionally, vesicular nanosystems can have a variety of shapes, such as spherical liposomes or tubular micelles, while nanocapsules are typically spherical or cylindrical in shape [48]. Overall, nanocapsules and other polymeric nanoparticles have many potential applications in drug delivery due to their ability to protect drugs and control their release profiles. They are an active area of research in the field of nanomedicine [48].

Finally, five studies developed liposomes as formulations, and the method for obtaining them was sonication [21,22,23,24,25]. Liposomes are vesicular structures with an aqueous interior bounded by one or more concentric lipid bilayers that may contain cholesterol to increase rigidity. Liposomes have numerous advantages as a drug delivery system, including their ability to encapsulate both hydrophilic and hydrophobic agents, increase drug loading, and protect encapsulated agents from metabolic processes in the body [48]. In this context, liposomes are more appealing to improve the viability of active substances via the topical route, as in the studies that used chamomile extract and *Santolina insularis* [10,25]. In addition, liposomes can enhance therapeutic effects, as in studies that evaluated the antibacterial activity of essential oil encapsulated in an *Artemisia afra* polymeric liposome [24], as well as studies of anti-inflammatory and antinociceptive activity using *Baccharis dracunculifilia* and *A. millefolium,* respectively [26,27].

Regarding the methods used for the physical-chemical analysis, the most used was dynamic light scattering, also known as photon correlation spectroscopy. This technique measures the size of various materials with diameters between 0.3 nm and 5.0 μm, such as pharmaceutical dispersions and emulsions, nanoparticles, micelles (surfactants), liposomes, colloids, and vesicles. It also allows measuring the zeta potential with good sensitivity, precision, and resolution. These measurements are necessary to determine the stability of emulsions and formulations as well as the characterization of nanoparticulate drug delivery systems [47]. All papers evaluated the particle size of the nanoformulations. The particle size information is essential to characterize whether the product is nano or not. However, some parameters, such as zeta potential and the polydispersity index (PDI), were absent in some papers. Among the formulations developed, 10 articles did not report the zeta potential or the PDI. The zeta potential is a measure of the electrostatic charge on the surface of particles in a colloid or suspension. It is defined as the potential difference between the surface of the particle and the surrounding fluid or medium. The zeta potential is influenced by the chemical and physical properties of the particle surface, as well as the composition of the surrounding fluid. The repulsion forces are sufficient to overcome the van der Waals attraction forces, preventing the occurrence of flocculation [49].

Among the general advantages of nanosystems are the increase in drug solubility, promotion of absorption, and protection against degradation of the active substance [48]. Comparing the nanosystems herein developed, we know that nanoemulsions are colloidal dispersions with higher stability than liposomes but are less stable than lipid nanoparticles. An essential disadvantage of nanoemulsions involves the use of surfactants in their composition, which renders this nanosystem more cytotoxic than liposomes or lipid nanoparticles. Nanoparticles are stable, easy to formulate and use, and biocompatible materials [48,50]. There are also polymeric nanoparticles with a lower acquisition cost than polymers and phospholipids, in addition to their greater stability and durability, which can facilitate storage at room temperature and increase the drug’s shelf life [50]. Liposomes are also biocompatible, as they are made of phospholipids, and their main advantage is the possibility of encapsulating hydrophilic and hydrophobic diagnostic or therapeutic agents [48]; however, obtaining phospholipids makes the process more expensive.

Although science is advancing in research, it is essential to note that the development of a product precedes legislation that guides the types of tests required and the necessary results of these tests. The Brazilian legislation on nanotechnology is nonexistent, and there are no definitions or terminology for nanotechnology. There are no agreed-upon protocols for toxicity testing of nanoparticles, and there are no standardized protocols to assess their environmental impacts. On the other hand, there is increasing marketing of nanotechnology products, thus denoting the need for safety assessments and mitigating potential health and environmental impacts [51].

### 2.4. Patents Targeting Products with Asteraceae Plants

A search for clinical trials and patents related to nanoformulations developed from *Artemisia*, *Achryrocline*, and *Matricaria* has been carried out. Although no clinical trials were found, four patents have been registered in the Derwent database using a combination of the keywords: *Artemisia*, *Matricaria*, *Achryrocline*, nanoemulsion, liposome, and nanoparticle. The number of patents found is low compared to the number of articles found in the review may indicate that the research in this field is still in its early stages, and there is a need for further research to validate the findings. Patents are not always a reliable indicator of research activity in a particular field. Some researchers may choose not to patent their findings for various reasons, such as the cost of patenting, the complexity of the patent application process, or concerns about the potential for patent infringement. On the other hand, a lack of tax incentives, standardized cultures for plant collection, and clear legislation are issues that can hinder the progress of research and development of nanoformulations using natural products. Nevertheless, the discovery of these patents can provide valuable insights for further research and development of nanoformulations based on these plant genera. It is important to continue exploring the potential of natural products in the development of nanoproducts, as they can provide safe and effective alternatives to synthetic drugs. Table 6 summarizes the patents found.

## 3. Conclusions

The search for new substances in biodiversity is a very much explored area because it presents alternatives for developing new drugs. The development of nanoformulations emerges as a tool for the delivery of pharmacologically active ingredients. All the studies evaluated showed an improvement in the pharmacokinetic parameters in the delivery of extracts and essential oils in all types of nanoformulations developed. The most prepared nanoproducts were nanoemulsions, and essential oils were the most used vegetable raw material. It is noticeable that the association between natural product chemistry and nanotechnology is still incipient, given the number of papers found in the literature. There is a concentration of studies in specific genera, such as *Achyrocline* and *Artemisia*. These genera have been studied for many years, and their therapeutic applications are already well established. Several biological activities were described and showed that progress is possible in several areas. For species of genus *Achyrocline*, antiprotozoal, antioxidant, hepatic protection, and antiviral activities were described. Regarding the genus *Artemisia*, antinociceptive, anticancer, antibacterial, larvicidal, and antifungal activities have been described.

Despite this association having promising results, there are several limitations in both areas—large-scale cultivation of plants, standardization of extracts, industrial scaling up in the development of nanoformulations, and absence of legislation on nanoproducts. There is no national regulation of nanoproducts. There are no agreed-upon protocols for nanoparticle toxicity testing or standardized protocols for assessing toxicity and environmental impacts. This lack of bureaucratic updating may be a factor preventing more nanoproducts from reaching the shelves.

From a future perspective, it is necessary to continue encouraging basic research and updating legislation in health to support and provide security in developing these products. The development of protocols and toxicity studies on nanosystems can contribute to the increase in primary, in vivo, and clinical trial research that depends on robust protocols to guarantee the safety of the assays. These modifications may reflect the incentive of investments in nanotechnology by private companies, making the nanoproducts to be developed.

## Figures and Tables

**Table 2:** Phytochemical characteristics of the species used in the development of the formulations.

Species	Vegetal Part Used	Extraction Method	Extracted	Chemical Marker	Extract Characterization	References
***Achyrocline satureioides***	Inflorescences	Maceration	Ethanolic extract (80%)	3-*O*-methylquercetin	HPLC-UV	[17]
***Achillea fragantissima*** ***and Achillea santolina***	Aerial parts	Hydrodistillation	Essential oil	cis-thujone and 1,8 cineole	GC-MS	[22]
***Achillea millefolium***	Leave	Refluxed	Aquous extract	-	-	[26]
***Achyrocline satureioides***	Aerial parts	Maceration	Ethanolic extract (80%)	3-*O*-methylquercetin	LC-UV	[16]
***Achyrocline satureioides***	-	-	Essential oil (purchased)	α-Pinene	CG-FID	[30]
***Achyrocline satureioides***	-	-	Essential oil (purchased)	α-Pinene	CG-FID	[31]
***Achyrocline satureioides***	-	-	Essential oil (purchased)	α-Pinene	CG-FID	[32]
***Achyrocline satureioides***	Inflorescences	Maceration	Ethanolic extract (80%)	3-*O*-methylquercetin	HPLC-UV	[9]
***Artemisia absinthium***	Whole plant	Maceration	Ethanolic extract	-	GC-MS	[11]
***Artemisia afra***	-	Hydrodistillation	Essential oil	-	-	[24]
***Artemisia annua***	Whole plant	Steam distillation	Essential oil	β-selinene	GC-FID and GC/MS	[15]
***Artemisia aucheri***	Aerial parts	Maceration	Methanolic extract	-	-	[33]
***Artemisia dracunculus***	-	-	Essential oil (purchased)	*P*-Allylanisole	GC-MS	[19]
***Artemisia dracunculus***	-	-	Essential oil (purchased)	Estragole	GC-FID and GC/MS	[13]
***Baccharis dracunculifolia***	Aerial parts	Maceration	Ethanolic extract	-	-	[27]
***Baccharis reticularia***	Leaves	Hydrodistillation	Essential oil	*D*-limonene	GC-FID and GC/MS	[18]
***Calendula offinalis***	-	-	Powder and oil (purchased)	-	-	[28]
***Calendula offinalis***	-	Supercritical CO_2_ extract	-	-	-	[29]
***Carlina acaulis***	Root	Hydrodistillation	Essential oil	Carlina oxide	GC-MS	[14]
***Matricaria chamomilla***	Flower	Percolation	Aqueous extract	Apigenin	HPLC	[25]
***Matricaria chamomilla***	-	-	Essential oil (purchased)	-	-	[34]
***Parthenium hysterophorus***	Whole plant	Maceration	Methanolic extract	-	-	[23]
***Pterocaulon balansae***	Aerial parts	Maceration	Hexanic extract	5-methoxy-6,7-methylenedioxycoumarin	HPLC-PDA/UPLC-MS	[20]
***Santolina insularis***	Aerial parts	Steam distillation	Essential oil	β-phellandrene	GC-FID and GC/MS	[10]
***Silybum marianum***	-	-	Commercial extract	Silybin	HPLC-PDA/UPLC-MS	[21]
***Stenachaenium megapotamicum***	Leaves and flowers	Hydrodistillation	Essential oil	Fokienol	GC-MS	[12]

**Table 3:** Chemical markers of the species.

Chemical Marker	Species	Identification	References
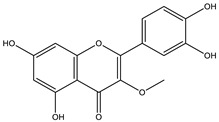	*Achyrocline satureioides*	HPLC-UV	[17]
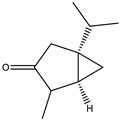	*Achillea fragantissima*	GC-MS	[22]
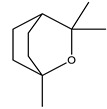	*Achillea santolina*	GC-MS	[22]
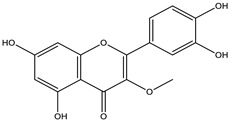	*Achyrocline satureioides*	LC-UV	[16]
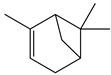	*Achyrocline satureioides*	CG-FID	[30]
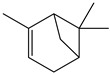	*Achyrocline satureioides*	CG-FID	[31]
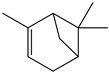	*Achyrocline satureioides*	CG-FID	[32]
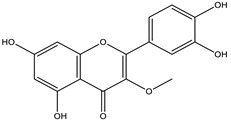	*Achyrocline satureioides*	HPLC-UV	[9]
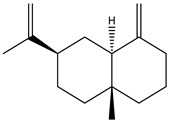	*Artemisia annua*	GC-FID and GC/MS	[15]
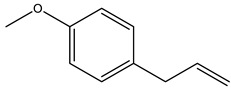	*Artemisia dracunculus*	GC-MS	[19]
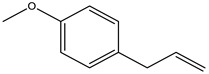	*Artemisia dracunculus*	GC-FID and GC/MS	[13]
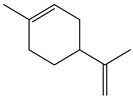	*Baccharis reticularia*	GC-FID and GC/MS	[18]
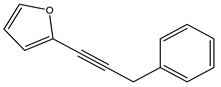	*Carlina acaulis*	GC-MS	[14]
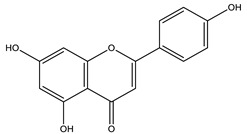	*Chamomile*	HPLC	[25]
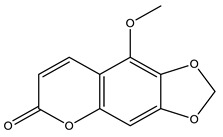	*Pterocaulon balansae*	HPLC-PDA/UPLC-MS	[20]
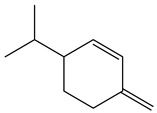	*Santolina insularis*	GC-FID and GC/MS	[10]
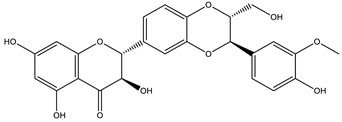	*Silybum marianum*	HPLC-PDA/UPLC-MS	[21]
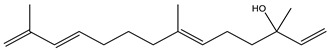	*Stenachaenium megapotamicum*	GC-MS	[12]

**Table 4:** Biological activities and assays performed for each formulation developed.

Nanoemulsion					
Species	Biologic Activity	Assay	Vector/Microorganism	Result	References
***Achillea fragantissima***	Acaricidal	Fumigant acaricidal activity	*Tyrophagus putrescentiae*	LC50 = 4.7 μL/L (3.2 ± 6.8)	[22]
***Achillea santolina***				LC50 = 9.6 μL/L (7.1 ± 13.3)	
***Achyrocline satureioides***	Antiviral	Antiherpes activity	Herpes Simplex Virus type 1 (HSV-1/KOS strain	IC50 = 1.40 ± 0.88 μg/mL	[16]
***Achyrocline satureioides***	Antioxidant	TBA-RS	-	77.6% inhibition of lipoperoxidation	[17]
***Achyrocline satureioides***	Antioxidant	TRAP	-	Six-fold higher reduction in the chemiluminescence	[9]
***Artemisia annua***	Antibacterial and antifungal	MIC	*Escherichia coli*	1.68 ± 0.72 µg/mL	[15]
			*Staphylococcus aureus*	1.62 ± 0.37 µg/mL	
			*P. aeruginosa*	1.46 ± 0.22 µg/mL	
			*S. pyogenes*	3.15 ± 0.16 µg/mL	
			*S. pombe*	2.01 ± 0.46 µg/mL	
			*C. albicans*	3.62 ± 0.65 µg/mL	
			*C. tropicalis*	4.29 ± 0.82 µg/mL	
			*C. dubliniensis*	3.63 ± 0.57 µg/mL	
			*C. krusei*	3.79 ± 0.57 µg/mL	
***Artemisia dracunculus***	Larvicidal	Larvicidal activity	*Anopheles stephensi* (3rd and 4th instar larvae)	82% of mortality	[19]
***Artemisia dracunculus***	Antibacterial	MIC and MBC	*Escherichia coli*	5.75/6.25 µg/mL	[13]
			*Listeria monocytogenes*	3.25/3.75 µg/mL	
			*Salmonella enteritidis*	4.75/5.75 µg/mL	
			*Shigella dysenteriae*	3.80/4.45 µg/mL	
			*Staphylococcus aureus*	2.5/3.25 µg/mL	
	Antioxidant	DPPH	-	IC50 = 0.052 mg/mL	
		FRAP		70.15 ± 0.63 µmol/mL of antioxidant capacity in the concentration of 10µg/mL	
***Baccharis reticularia***	Larvicidal	Larvicidal activity	*Aedes aegypti*	LC50 = 221.273 µL/mL (24 h); LC50 = 144.685 µL/mL (48 h)	[18]
***Carlina acaulis***	Larvicidal	Larvicidal activity	*Lobesia botrana (1st instar larvae)*	LC50 = 9.04 µL/mL; LC90 = 17.70 µL/mL	[14]
***Parthenium hysterophorus***	Herbicide	Seed germination bioassay	*Diodia ocimifolia*	100% of germination inhibition in the concentration of 5 g L^−1^	[23]
***Stenachaenium megapotamicum***	Antifungal	MIC	*Epidermophyton floccosum*	5.18 μg/mL	[12]
			*Trichophyton rubrum*	41.5 μg/mL	
**Polymeric Liposome**					
***Achillea millefolium***	Antinociceptive	Formalin test	Male Wistar rat (220–280 g) Approved from Ethical Committee	66% of pain inhibition	[26]
***Artemisia afra***	Antimicrobial	MIC	*E. coli*	270 μg/mL	[24]
			*P. aeruginosa*	>270 μg/mL	
			*S. aureus*	>270 μg/mL	
			*C. albicans*	17 μg/mL	
***Baccharis dracunculifolia***	Anti-inflammatory	Zymosan-induced joint inflammation	Male Wistar rat (180–200 g) Approved from Ethical Committee	Decreased joint swelling and inflammatory interleukins	[27]
***Chamomile***	Anti-inflammatory	Clinical trial	Human being (24–65 years old) Approved from Ethical Committee	Reduction in erythema, edema, vesicular, and excoriation	[25]
***Santolina insularis***	Skin permeation	-	-	Improvement of the active permeation delivering in the skin	[10]
**Polymeric Nanoparticles**					
***Achyrocline satureioides***	Antiprotozoan (*Trypanosoma evansi*)	Hematological analysis in vivo	Female Wistar rat (203 g average) Approved from Ethical Committee	The treatment controls the infection but does not eliminate it.	[30]
***Achyrocline satureioides***	Antioxidant *(Trypanosoma evans* infection)	TBARS	Female Wistar rat (200 g average) Approved from Ethical Committee	The treatment with avoided the increase in ROS and TBARS levels of infected rats.	[31]
***Achyrocline satureioides***	Hepatic protection *(Trypanosoma evans* infection)	MTT assay	Female Wistar rat (200 g average) Approved from Ethical Committee	Increase the hepatic protection against the infection and reduces cytotoxic damage in liver	[32]
***Artemisia aucheri***	Antinociceptive	Formalin test	Male Sprague-Dawley rats (260–300 g)	Bupivacaine in combination with *A. aucheri* gave a synergic effect in antinociceptive activity	[33]
***Artemisia absinthium***	Anticancer	MTT assay	MCF-7; MDA MB-231	IC50 = 176.83/181.39 µg/mL	[11]
***Calendula officinalis***	Anticancer	Cytotoxicity assay	Human breast adenocarcinoma MCF7	Improvement of anticancer effects	[28]
***Calendula officinalis***	Wound healing	Delivery study	-	Improve epithelium repair in ocular surface and works as good delivery system	[29]
***Matricaria chamomilla***	Antileshmaniasis	Antiproliferative assay	*Leshmaniasis amazonensis*	IC50 = 3.33 µL/mL	[34]

TBARS = thiobarbituric acid reactive substance assay; TRAP = total reactive antioxidant potential; TAR = total antioxidant reactivity; MIC = minimum inhibitory concentration; MBC = minimum bactericidal concentration; DPPH = 2,2-Diphenyl-1-picrylhydrazyl; FRAP = ferric reducing ability of plasma; CAT = catalase; SOD = superoxide dismutase (SOD); GSH = glutathione reduced; MTT = 3-(4,5-dimethylthiazol-2-yl)-2,5-diphenyltetrazolium bromide.

**Table 5:** Physicochemical characteristics of nanoformulations.

Formulation	Species	Obtaining Method	Physicochemical Characterization	Method	Result	References
Nanoemulsion	*Stenachaenium. megapotamicum*	Spontaneous emulsification	Particle size	PCS (photon correlation spectroscopy)	210 nm	[12]
			PDI		0.369	
			Zeta potential		-	
Nanoemulsion	*Achyrocline satureioides*	Spontaneous emulsification	Particle size	PCS	237.35 ± 12.71nm	[16]
			PDI		0.09 ± 0.04	
			Zeta potential		−42.45 ± 1.96 mV	
Nanoemulsion	*A. satureioides*	Spontaneous emulsification	Particle size	DLS	295.6 ± 9.0nm	[17]
			PDI		0.211 ± 0.021	
			Zeta potential		−43.6 ± 2.1 mV	
Nanoemulsion incorporated into hydrogel	*A. satureioides*	Spontaneous emulsification	Particle size	PCS	246.8 ± 3.3 nm	[9]
			PDI		0.22 ± 0.1 nm	
			Zeta potential		−42.5 ± 2.2 mV	
Nanoemulsion	*Baccharis reticularia*	Non-heating and low energy method	Particle size	PCS	94.5 ± 1.9 nm	[18]
			PDI		0.382 ± 0.048	
			Zeta potential		−21.5 ± 1.4 mV	
Nanoemulsion	*Artemisa draucunlus*	Spontaneous method	Particle size	DLS	11.20 ± 1.10 nm	[19]
			PDI		2.1 ± 0.08	
Nanoemulsion	*Pterocaulon balansae*	Spontaneous emulsification	Particle size	DLS	276 ± 54 nm	[20]
			PDI		0.215 ± 0.09	
			Zeta potential		−21.5 ± 5.9 mV	
Nanoemulsion	*Silybum marianum*	Stirred for 24 h at 25 °C	Particle size	DLS	20.09 ± 0.04 nm	[21]
			PDI		0.059 ± 0.014	
			Zeta potential		−6.63 ± 1.73 mV	
Nanoemulsion	*Achillea fragantissima* and *A. santolina*	High energy (ultrasonication)	Particle size	DLS	91.3 ± 9.6 nm/104.6 ± 14.1	[22]
			PDI		0.20 ± 0.02/0.26 ± 0.01	
			Zeta potential		-	
Nanoemulsion	*Parthenium hysterophorus*	High energy emulsification	Particle size	DLS	218 nm	[23]
			PDI		0.08	
			Zeta potential		−26.80 mV	
Nanoemulsion	*Artemisia draucunlus*	Ultrasound at ambient temperature	Particle size	DLS	50 nm	[13]
			PDI	-	-	
			Zeta potential	Electrophoretic mobility	−30mV	
Nanoemulsion	*Carlina acaulis*	High-pressure homogenizer	Particle size	DLS	143.9 nm	[14]
			PDI		0.28 ± 0.005	
			Zeta potential		153.93 ± 1.58	
Nanoemulsion	*Artemisia annua*	Sonication	Particle size	DLS	160 ± 2.2	[15]
			PDI		0.041	
			Zeta potential		-	
Liposome	*Artemisia afra*	Sonication	Particle size	DLS	8.269 ± 0.796 μm	[24]
			PDI		0.429 ± 0.053	
			Zeta potential		9.0 ± 2.40 mV	
Liposome	*Matricaria chamomilla*	Stirring in ultrasound bath	Particle size	DLS	304 nm	[25]
			PDI		0.14	
			Zeta potential		-	
Liposome	*Santolina insularis*	Ultrasonication	Particle size	DLS	111 ± 7 nm	[10]
			PDI		0.15	
			Zeta potential		−17 ± 2 mV	
Liposome nanoparticle	*Achillea millefolium*	Ultrasonication	Particle size	DLS	160 nm	[26]
			PDI		0.25	
			Zeta potential		−30 to −60 mV	
Liposome	*Baccharis dracuncufolia*	-	Particle size	Laser light scattering	182.5 ± 9.2	[27]
			PDI		0.13 ± 0.05	
			Zeta potential		−0.08 ± 0.26 mV	
Nanoparticle	*Calendula officinalis*	Stirring	Particle size	Scanning electron microscope	541.68 ± 85.14 nm	[28]
			PDI		-	
			Zeta potential		-	
Solid lipid nanoparticle	*Calendula officinalis*	Warm microemulsion technique	Particle size	PCS	80 nm	[29]
			PDI		-	
			Zeta potential		−35 to 48 mV	
Nanocapsule	*Achyrocline satureioides*	Deposition of preformed polymer	Particle size	DLS	256.6 ± 1.27 nm	[30]
			PDI		0.097 ± 0.007	
			Zeta potential		−30 ± 0.18 mV	
Nanocapsule	*Achyrocline satureioides*	Deposition of preformed polymer	Particle size	DLS	235.9 nm	[31]
			PDI		0.112	
			Zeta potential		−29.3 mV	
Nanocapsule	*Achyrocline satureioides*	Deposition of preformed polymer	Particle size	DLS	235.9 nm	[32]
			PDI		0.112	
			Zeta potential		−29.3 mV	

**Table 6:** Nanoproducts patents from Asteraceae plants.

Patent Number	Species	Nanoformulation	Indication	References
CN1368306-A	*Artemisia* sp.	Nanoparticle	Antinociceptive formulation	[52]
MX2011013407-A1	*Matricaria recutita and Calendula officinalis*	Liposome	Phthalmic solution used for preventing and combating dry eyes	[53]
CN105017913-A	*Artemisia argyi*	Liposome	Antibacterial resin used for environmentally friendly paint	[54]
CN102552414-A	*Matricaria chamomilla*	Nanoemulsion	Acne, eczema, psoriasis, skin inflammation, pustule, and wound infection.	[55]

## Data Availability

All data will be available under request.
